# Barriers and strategies for primary health care workforce development: synthesis of evidence

**DOI:** 10.1186/s12875-024-02336-1

**Published:** 2024-03-27

**Authors:** Aklilu Endalamaw, Resham B Khatri, Daniel Erku, Anteneh Zewdie, Eskinder Wolka, Frehiwot Nigatu, Yibeltal Assefa

**Affiliations:** 1 School of Public Health, The University of Queensland, Brisbane, Australia; 2 College of Medicine and Health Sciences, Bahir Dar University, Bahir Dar, Ethiopia; 3 Health Social Science and Development Research Institute, Kathmandu, Nepal; 4 Centre for Applied Health Economics, School of Medicine, Griffith University, Brisbane, Australia; 5 International Institute for Primary Health Care in Ethiopia, Addis Ababa, Ethiopia

**Keywords:** Health care provider, Health Workforce, Primary Health Care

## Abstract

**Background:**

Having a sufficient and well-functioning health workforce is crucial for reducing the burden of disease and premature death. Health workforce development, focusing on availability, recruitment, retention, and education, is inseparable from acceptability, motivation, burnout, role and responsibility, and performance. Each aspect of workforce development may face several challenges, requiring specific strategies. However, there was little evidence on barriers and strategies towards comprehensive health workforce development. Therefore, this review explored barriers and strategies for health workforce development at the primary health care level around the world.

**Methods:**

A scoping review of reviews was conducted following the Preferred Reporting Items for Systematic Reviews and Meta-analysis Extension for Scoping Reviews. The article search was performed in Google Scholar, PubMed, Web of Science, and EMBASE. We used EndNote x9 for managing the collected articles, screening processes, and citation purpose. The scoping review included any kind of review articles on the application of health workforce development concepts, such as availability, recruitment, retention, role and responsibility, education and training, motivation, and burnout, with primary health care and published in English anywhere in the world. Based on the concepts above, barriers and strategies for health workforce development were identified. The findings were synthesized qualitatively based on the building blocks of the health system framework. The analysis involved specific activities such as familiarization, construction of the thematic framework, indexing, charting, and interpretation. The results were presented in texts, tables, and figures.

**Results:**

The search strategies yielded 7,276 papers were found. Of which, 69 were included in the scoping review. The most frequently cited barriers were financial challenges and issues related to health care delivery, such as workloads. Barriers affecting healthcare providers directly, including lack of training and ineffective teamwork, were also prominent. Other health system and governance barriers include lack of support, unclear responsibility, and inequity. Another notable barrier was the shortage of health care technology, which pertains to both health care supplies and information technology. The most common cited effective strategies were ongoing support and supervision, engaging with communities, establishing appropriate primary care settings, financial incentives, fostering teamwork, and promoting autonomous health care practice.

**Conclusions:**

Effective leadership/governance, a robust health financing system, integration of health information and technology, such as mobile health and ensuring a consistent supply of adequate resources are also vital components of primary health care workforce development. The findings highlight the importance of continuous professional development, which includes training new cadres, implementing effective recruitment and retention mechanisms, optimising the skill mix, and promoting workplace wellness. These elements are essential in fostering a well-trained and resilient primary health care workforce.

**Supplementary Information:**

The online version contains supplementary material available at 10.1186/s12875-024-02336-1.

## Background

Health workforce is a core component of health system performance [1]. It is essential for achieving universal health coverage (UHC), health security, and socio-economic development [2, 3]. The availability of well-organised health workforce is crucial for increasing accessibility of services, reducing healthcare costs, and preventing morbidity and premature mortality [4, 5]. Health workers play a significant role in the dynamic global system, diverse population and emerging pandemics by strengthening health system [6]. For example, the COVID-19 and Ebola outbreak response revealed the substantial importance of health care providers [7, 8]. This implies that health workers can help prevent, detect, and respond to epidemics and outbreaks, which supports the metaphor that ‘there is no health without a workforce’ [9], but health workers need appropriate health workforce development.

Health workforce development is the process of planning, training, deploying, and retaining healthcare professionals [10]. It involves improving health workers’ quality, performance, skills, and availability. United Nations (UN) and World Health Organisation (WHO) advised that every country’s health system should regulate human resource development for health [11–13]. Health workforce development also involves assessing the current and future health workforce needs and gaps; developing and implementing policies and strategies to address the health workforce challenges; strengthening the education and training of health workers to ensure they are competent, fit-for-purpose, and fit-for-practice; enhancing the management and governance of the health workforce; promoting the health and well-being of health workers and creating a supportive work environment; and understanding roles and responsibilities [14–16].

Evidence is needed to identify the barriers to health workforce development and the available strategies to address them, to fill the observed gaps [17–19] and support the progress towards achieving UHC [20]. Available articles are presented in a fragmented way for specific aspect. For example, studies examined factors in recruiting rural pharmacists and task shifts of health care providers [21, 22]. The WHO Global Health Observatory, which served only as a repository mechanism to show the number of health cadres, has also presented the number of health care cadres [23]. However, Available literature and WHO health observatory did not provide barriers to and strategies for developing health workforce.

The current review explored two research questions: (1) what barriers do primary health care (PHC) settings face in developing a health workforce, and (2) what strategies can address these barriers and enhance workforce development?

## Methods

### Protocol

The scoping review was conducted based on Arksey and O’Malley framework’s components, including identifying the research question (mentioned at the end of the background section), searching relevant studies, selecting eligible studies, extracting the data from the included studies, summarising and reporting the results, and conducting consultation (optional) [24]. This review has been notified following the preferred reporting items for systematic reviews and meta-analysis extension for scoping reviews [25].

### Eligibility criteria

The scope of the current review was determined by applying the population, context, and concept framework. Therefore, based on Pollock et al’s recommendations [26], the population or participants was health care providers; context is operationalised based on study type and study settings, which are any reviews in study types and primary health care level around the world regarding geography. Concepts are related to various aspects of health workforce development, such as education and training, role and responsibility, recruitment, retention, availability, burnout, motivation, acceptability, and performance. These dimensions contributed to workforce development.

We included and evaluated articles that addressed the barriers and strategies for at least one of the dimensions of health workforce development in PHC settings. We included any reviews published in English before 04 July 2022 because this was the last search date. We did not limit our search to a specific inception date range, but the search strategy in PubMed displayed that the first article published was in 1975. During a preliminary search, we ensured the presence or absence of literature that addressed our research questions comprehensively. Based on our preliminary search, we recognised that there are different reviews for a single dimension related to health workforce development. Therefore, we decided to focus on a review article and gathered relevant findings from it. We have extracted the main findings from the included reviews. We refer to the primary studies of the included reviews only when we find that the reported findings in the review are unclear and create confusion among us.

### Search strategy and study selection

We searched for literature using Google Scholar, PubMed, EMBASE and Web of Science databases. We combined key terms using “AND” or “OR” Boolean operators and asterisk (*). The search terms are “health care”, “primary healthcare”, “primary care”, “work force”, workforce, worker, person, professional, “human resource for health”, availability, accessibility, distribution, location, acceptability, coverage, quality, performance, education, training, development, deployment, composition, skill-mix, recruitment, staffing, turnover, retention, motivation, burnout, role, responsibility, and review. Using key terms, the search strategies were conducted on the databases. Then, filters were applied to include review articles published in English. The first author (AE) developed a search strategy in consultation with the last author (YA). Other co-authors (RKB and DE) commented on it during weekly regular meetings. The detail search strategy for each database is presented in supplementary file 1. The search results were exported into EndNote X9 for duplication removal and managing the article screening process. The first author (AE) exported all available articles into the EndNote reference manager. Then, AE performed an article duplication check and removed duplicated articles. Following comments from RBK, DE, and YA on the weekly meeting, AE conducted title and abstract screening, followed by full-text screening, to include the eligible articles for the data extraction process.

### Data extraction and synthesis

Data extraction format was prepared using Microsoft Excel. The first author and year of publication, country represented by included article, number of articles included in each included review, type of review, and main findings were extracted. First, the included article was examined to understand which specific domain it included. Second, barriers and strategies were synthesised for each identified domain. The findings were synthesized qualitatively based on the building blocks of the health system framework. The analysis involved specific activities such as familiarization, construction of the thematic framework, indexing, charting, and interpretation. The results were presented in texts, tables, and figures. PHC workforce development domains allowed for easy description, categorisation and plotting of the extracted data. Finally, strategies were framed based on the health system functions related to the health workforce. Health system functions are called building blocks of health system, which include health workforce development as part of them. The other elements are leadership and governance, service delivery, health system financing, health information systems, and medical supplies and information technologies [1].

## Results

### Search results

**Fig. 1:**
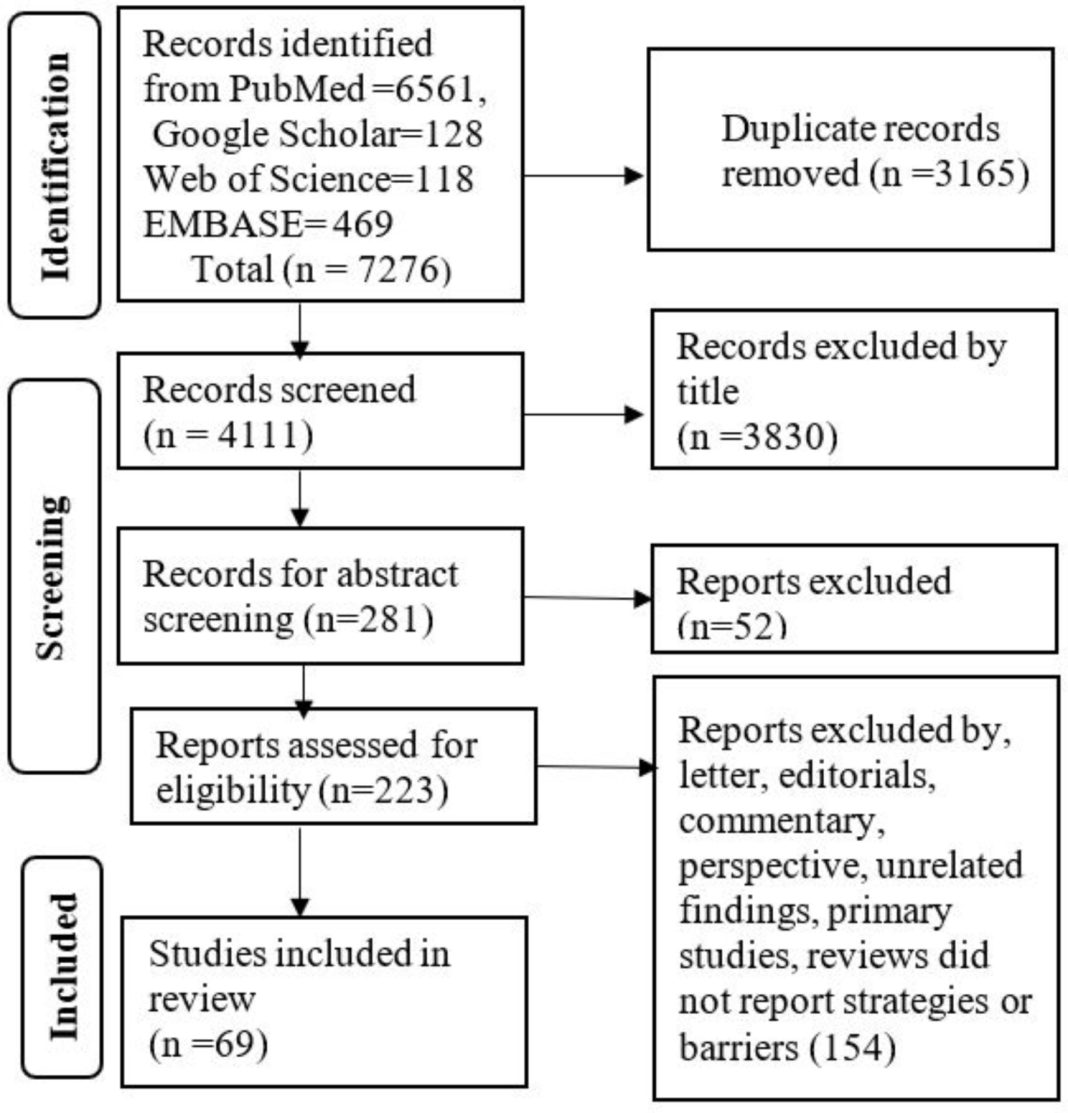
Article selection flow chart

The search identified a total of 7,276 articles. After duplicates were removed, 4,111 articles remained. Then, 3,830 records were excluded by title and 281 were screened for abstract review. After excluding 52 articles during it, 223 were eligible for full-text review. Finally, 154 articles were excluded, and 69 were included (Fig. 1). The included articles, as named initially, were classified into the following types: one realist review, one synthesis of qualitative research, one umbrella review, four narrative reviews, four scoping reviews, seven literature reviews, seven reviews, eight integrative reviews, eight systematic reviews with meta-analysis, and twenty-eight systematic reviews. These studies reviewed data from many countries across regions of Africa, America, Europe, the Eastern Mediterranean, Southeast Asia, and the Western Pacific. Supplementary file 2 shows the characteristics of the included articles.

### Barriers and strategies for PHC workforce development

This section presents a narrative synthesis of barriers to health workforce development and strategies. Finally, a strategic framework is presented based on the building block of health system. The frequently reported barriers are lack of training, ineffective teamwork, ambiguity of responsibility, health financing problems, and inequity. These barriers, along with professional interest and perception, unwelcoming health care environment, lack of recognition, and shortage of health care technology, are presented in the following texts.

### Health system governance and leadership-related barriers

Lack of recognition to the health care providers, lack of clear responsibility, and unwelcoming working environment were barriers to health workforce development.

Lack of recognition can include a lack of support (e.g., emotional and practical) for professionals, leading to lack of service acceptability in the UK, Australia, Holland, and New Zealand [27–29]. This includes lack of family support, lack of recognition, lack of non-financial incentive, non-rewarding opinion or lack of positive community opinion, which demotivate health professionals in China, the USA, Australia, and Canada [30, 31]. Lack of recognition [32] and lack of support [33] increase health worker’s turnover in the UK. It also results in inadequate staffing in the same country [32].

The absence of clear responsibility affects health workforce development. This includes inconsistent definitions of the profile of community nurses in Australia [34] and role ambiguity in rural settings (not specific to a single country) [3]. These factors affect the retention of professionals. In addition, the difficulty of expanding scopes of practice has been a challenge for allocating new cadres in the USA [35].

Unwelcoming health care environment can be also related to health system governance-related barriers. Health professionals were unwilling to join unsuitable working environments in Australia [36]. Conflict zone and workplace violence affect the proper recruitment and retention of health workers [3] and increase burnout among health workers in the USA and elsewhere [37, 38]. Moreover, poor health care infrastructure demotivates health workers in the USA, Australia, and Canada [30]. Rigid career structures push providers to leave their profession or workplace in the UK [32].

### Health financing related barriers

Financial problems hinder the availability, acceptability, recruitment, role and responsibility, and education of health professionals. Financial challenge is one of the common barriers to PHC workforce development in different countries, such as the UK, the USA, Australia, Holland, Asia, Europe, and Central America [27, 36, 38–40]. It also makes it challenging to implement proper recruitment and retention of health care providers [18]. Financial challenge or resource inadequacy prevents health professionals from fulfilling their roles and responsibilities [36] and causes burnout among health care providers [37, 38, 41].

### Health worker related barriers

The common barriers were the lack of continuous professional development opportunities, lack of training, and unfair distribution. The continuous development for the health workforce (training and education) as a barrier was identified in different countries. These include the UK [27, 29], Australia [27, 42], Holland [27], New Zealand [27], and China [17, 31], as well as others that are not linked to a single country [3, 19, 43]. The absence of training or career prospects health professionals’ turnover rates [19]. Recruitment [3], acceptability of health care practice [27], service provision [29] and performance of health care providers [31] are influenced by lack of educational preparation. Compared to those who received their education in their own country (in Australia), general practitioners who trained overseas tended to leave their workplace more likely [42]. Due to these barriers, a large percentage of PHC workers had turnover intention [17, 43].

Teamwork problem was another barrier. Health professionals could not practice properly due to the absence of teamwork and team with poor communication in the United Kingdom (UK) [29]. A large percentage of health professionals developed burnout in the United States of America (USA) and other countries [37, 38].

Unfair distribution of health workers was also a crucial barrier. People living in rural (underserved) areas suffer from lack of physicians and health professional teachers in Central America [39]. Health care providers’ intention to live in geographically remote areas in Australia [42], attitudes towards rural practice and scarcity of resources in rural setting in the USA and elsewhere increase the gap in the availability of health workers [44, 45].

Interest and perception are other factors. Lack of interest in medical education in Australia [36] affects recruitment and overall health workforce development. Lack of intrinsic job satisfaction, self-inefficacy and lack of self-motivation affect health care providers’ motivation in the USA, Australia, Canada, and America [30, 38] and increases burnout among health workers in the USA and elsewhere [38, 41].

Confusion and self-doubt also affect the allocation of professionals, based on an integrative review [3]. Health professional’s doubt about clinical relevance hinders the establishment of workforce development due to a lack of acceptability (not specific to a single country) [46].

### Health care delivery related barriers

High workload is the most frequently identified barrier. The workload in the UK, Australia, Holland, and New Zealand [27] and time restraint in the UK [29] are constraints for the acceptability of services. It also demotivates health workers in Australia, Canada, and America [30] and causes burnout among health care providers in the UK, low-and middle-income countries, and elsewhere [37, 38, 47]. Additionally, high workload in the UK, the USA, China, and elsewhere [17, 19, 32, 33, 45] and long working hours in the UK, the USA, and elsewhere [32, 44, 45] enforce health workers to leave their workplace or profession. Others are patients with low adherence to care and problems with guidelines [29].

### Health care supplies and information technology related barriers

The emerging technology can have negative effects on health care providers. It can increase burnout among health care providers. One example of how emerging technology can cause burnout is the transition phase to electronic health records in the USA [38]. In addition to this challenge, health care providers face data incompatibility issues with different electronic health record systems and unclear legal liability to remote technology services in different countries [46]. Insufficient information technology support also affects health workforce development [29]. Scarcity of supplies was another barrier [38, 45].

### Sociodemographic related barriers

Sociodemographic contexts are demographic and economic factors. Female health workers in the USA were not willing to work far away [35, 36]. Others, such as younger age [43], highly educated [43], and senior staff [17] were less likely to retain in their workplace. In addition, burnout was higher among health workers with low economic conditions and smokers [41]. Physician race/ethnicity and language influenced the availability of health workers [18].

### Strategies

Maintaining an adequate health workforce in PHC requires effective strategies. The main strategies are related to appropriate health financing, availing clear roles and responsibility, conducting proper supervision and leadership, offering continuous education and training for the staff, providing recognition for the health workers, establishing a welcoming work environment, presence of community engagement and collaboration, availing effective teamwork, and proper utilisation of information technology.

### Health system governance and leadership-related strategies

Proper supervision and leadership were effective strategies. Health system governance roles have played a significant role in health workforce development. For instance, strong leadership in Australia, Canada, New Zealand, and the USA [48–50], ongoing support and supervision in low-, middle-, and high-income countries [2, 22, 27, 28, 48, 50–53]. Furthermore, continuous supervision and effective leadership enhance the retention of health care workers in Australia [50]. In addition, effective leadership and partnership were effective strategies in the USA [49].

Community engagement and collaboration were effective strategies. One of the strategies to improve the recruitment and retention of PHC providers in rural and underserved settings is to involve various sectors and communities in the process. Sectors such as educators and policymakers can collaborate with local health facilities and organizations to provide training, support, and incentives for PHC providers. Communities can also participate in the recruitment and retention of PHC providers by offering social and cultural integration, feedback, and recognition. Therefore, the involvement of sectors and communities can enhance the attractiveness and sustainability of rural and underserved practice for PHC providers. This strategy has been implemented and evaluated in several countries, such as Australia, Ukraine, Canada, the USA, and New Zealand [21, 50, 54].

Clear roles and responsibilities can be part of determining the scope of practice. Countries (in Australia, the USA, and elsewhere) adopted various PHC providers with clear job descriptions [55–57]. Most are working in health promotion and disease prevention (including early screening) in Southeast Asia region, the Americas, Europe, and Australia [58–61]. Some professionals are designed to perform specific roles. For instance, physician’s assistant provides supplemental medical services, takes health history, prescribes diagnostic tests and referrals, conducts medical diagnosis, manages acute medical problems, and provides comprehensive care, including consultation, especially for underserved rural populations in the USA, Saudi Arabia, the UK, Canada, and Australia [62–65]. Advanced nurse practitioners perform health assessment, diagnosis, ordering tests, health promotion, disease prevention and wellness services, sexual health and maternity services, provide care for chronic and acute problems, making referrals, providing outreach care for vulnerable and marginalised groups, teaching and researching, administrative and managerial roles, patient advocacy, undertaking a similar role with doctors or assisting doctors and work in Asia, Europe, Americas, Oceania, and Multiple regions [40]. General practitioners educate other healthcare professionals, lead and develop services, provide respiratory, dermatology, palliative and neurology care, and perform specific procedures in Australia [55]. The pharmacist’s role in general practice clinics was medication review in the USA, the UK, Canada, South America, and Asia [66]. Establishing a proper scope of practice in Australia, Ukraine, Canada, the UK, and New Zealand [21, 67], and autonomous practice promote recruitment in Australia, Ukraine, Canada, the UK, and New Zealand [21, 54]. Furthermore, expanding the scope of practice in Afghanistan and elsewhere [68, 69], easing restrictions on their scope of practice [69], and providing accessible care [3] increased the availability of health workers.

Recognition of staff also enhances workforce development. There have been other non-financial supports that enforce health workforce development. Recognition in Australia [50, 70], respecting health care providers [32], and non-financial incentives in low-and middle-income countries [71] are effective strategies for retaining health workers.

### Health financing related strategies

Appropriate management of healthcare financing is a crucial strategy for health workforce development. It keeps availability, improves recruitment, and enhances retention of health workers. Specific health financing strategies include financial incentives in the USA, the UK, Netherlands, Russia, Canada, Australia, China, Afghanistan, and low- and middle-income countries [22, 30, 31, 52–54, 67, 68, 71, 72], financial remuneration in the USA, Australia, and elsewhere [50, 54, 72], government funding in the Asia, Europe, Americas, Oceania, Australia, New Zealand, and across regions [40, 48], health care services with free of costs in Greece, the UK, Malaysia, Australia, Canada, the USA, Finland, Ireland, Sweden, Nepal, and Iran [73] and increase salaries for health care workers [74]. Financial support, including incentives facilitates effective recruitment in Canada, the USA, and elsewhere [54, 67, 72]. Additionally, an effective financial remuneration system improves the retention of health care providers in the USA, Australia, and elsewhere [50, 54, 72] and increases the availability of health workers in Afghanistan [68].

### Health worker related strategies

Training and education are fundamental for health workforce development.

Higher education institutions are providing training to undergraduate students, for example, in Colombia [75]. Different training approaches, such as theoretical practice care and case orientation, educational modules, clinical partner programs, seminars, expository courses, knowledge translation programs, theatre (simulation)-based training and toolkits, are effective in training PHC providers in Europe and North American continent [76]. Skill-mix and evidence-based education are required for the PHC service providers’ education in the USA [77]. Some strategies, like course-based training, mentoring and workshops, have been effective for palliative and end-of-life care providers to continuous professional development [78] and traditional didactic methods to teaching theoretical knowledge to doctors and nurses (during the viral epidemic) [79]. An institution has adequate providers during recruitment if it supports staff’s educational program, professional development, or research in high-income countries and elsewhere [19, 54, 80]. Providing rural-based undergraduate and postgraduate training and placement, was exceptionally important to recruit professionals for underserved areas in Canada, Australia, and elsewhere [67, 80, 81]. Educational opportunities (e.g., sub specialisation) is important for retention of health workers in Organisation for Economic Cooperation and Development countries [50, 70]. Availing medical education institutions in the USA [35], providing professional training in the USA, Afghanistan, and elsewhere [3, 35, 68] and introducing new cadres (e.g., physician assistants and community-based practitioners) in Afghanistan, the USA, and elsewhere [68, 69, 82] increase health workers availability. Moreover, role awareness and professional development are effective strategies in the USA [49].

### Health care delivery-related strategies

Effective teamwork, optimum working hours and peer support are important strategies. Teamwork and peer support have been identified as effective strategies for recruitment and health worker retention in different countries. In an institution where group practice or teamwork is available, smooth recruitment of health workers can be conducted in Australia, Ukraine, Canada, the USA, and New Zealand [21, 50]. In addition, teamwork is an effective strategy in the USA [49]. Additionally, strengthening communication within the health team is vital to establish well-functioning health workers [75]. Peer support also effectively promotes the retention of health workers in Australia [50]. Improving the work structure, allowing autonomous practice, and reducing working hours when nurses approach retirement is one way to create a work environment that retains health workers in the UK, the USA, Australia, and around the world [32, 43, 50, 54].

### Health care supplies and information technology related strategies

Clinicians, administrators, and other clinic staff have a positively view of remote monitoring technology integration into PHC [46]. Remote monitoring technology allows health workers to monitor and communicate with patients remotely, reducing the need for travel and increasing access to care. This also encourages the availability of health workers in rural areas [3]. Availability of equipment enhances health workforce development [52].

### Strategies towards improving sociodemographic contexts

Welcoming health care environment is crucial. Creating an attractive rural lifestyle for PHC providers who work in rural settings is important to retain them and recruit new personnel in Canada [67]. In addition, friends and family living in the PHC institution area can also influence the recruitment and retention of PHC providers in Australia, Ukraine, Canada, the United States of America, and New Zealand [72, 80, 81]. However, different strategies may be needed for different contexts and settings. For example, residential background-based recruitment (rural residence to rural PHC) [21]. Moreover, preparation for rural life and medicine, enhancing partner receptivity to rural living, and creating a balanced work-life are crucial for recruitment and retention [72].

#### Strategic framework

**Fig. 2:**
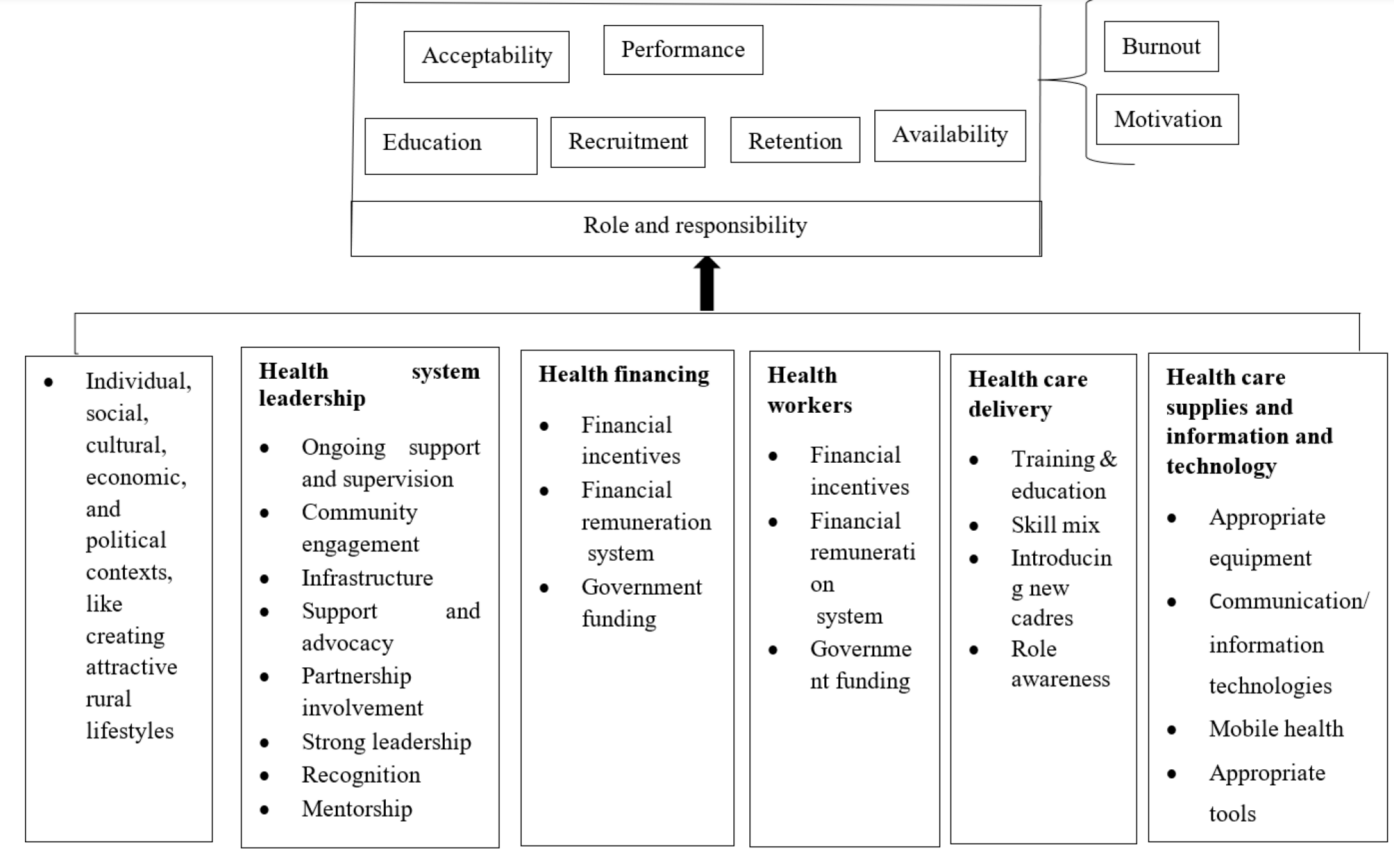
Strategic framework for PHC workforce development

Individual, social, cultural, economic, and political contexts influence primary health care workforce development. The characteristics of learners affect their persistence in health care providers’ education. The country’s political willingness and policy priority to enhance service delivery affect PHC workforce development. These factors include improving the quality of health providers’ training, continuous professional development, and the ability to pay salaries and incentives. Other strategies contributing to PHC workforce development are health financing, information technology, and supplies and equipment. Figure 2 illustrates the relationship between these factors and PHC workforce development (Fig. 2).

## Discussion

The main aim of this review is to identify the barriers and strategies for comprehensive PHC workforce development. Health policymakers or managers can use this evidence to design and implement effective interventions for PHC workforce development. Future researchers can utilise this review as a guiding framework to examine the PHC workforce development in any country.

Primary health care workforce development was facilitated by strong health leadership. Strong leadership is effective in strategic planning, organising resources, staffing, and managing service providers [83]. According to a previous survey, 84% of physicians and advanced practitioners need strong leadership to stay in their current profession [84]. Moreover, effective leadership helps to identify gaps, intervene in the shortage of resources, fill gaps in service providers, and provide timely feedback [85]. These facilitate the right support from the organisation, which positively impacts work engagement and job satisfaction [86]. Strong leadership, that entertains family and professional association support, improves the recruitment and availability of health care providers, and strengthens PHC workforce development [87]. Likewise, those PHC institutions that engaged communities and sectors encouraged health workforce development. Communities that shared their experiences explained that community engagement effectively recruited and retained health care providers [88]. Additionally, community engagement enhanced health workers’ motivation [89] and increased their performance [90, 91].

The impact of leadership on PHC workforce development has been demonstrated in different ways. Collaboration, teamwork, autonomous practice, and appropriate working environment enhanced PHC workforce development as leadership roles. A previous survey revealed that the majority (88.8%) of physicians believed that leadership is key in creating a collaborative environment [84]. This collaborative environment can foster teamwork that positively influences PHC workforce development. An institution which supports autonomous practice, allowing practitioners to practice their roles independently [92], has better achievement in PHC workforce development. This is in line with an integrative review that concluded that autonomous practice helps enhance retention and recruitment [93]. An effective leader can create an appropriate work environment. An appropriate health care environment that includes suitable and inclusive infrastructure, delivering safe and culturally appropriate service [94], results in job satisfaction [95]. Job satisfaction motivates PHC providers [53] and facilitates retention and recruitment [70]. Therefore, strong health leadership is essential for developing and sustaining a competent and motivated PHC workforce.

Financial supports facilitate PHC workforce development. Financial incentives increase acceptability, availability, performance, retention, recruitment, and motivation and prevent burnout. Another piece of literature draws the significant impact of financial support on public health workforce development to a conclusion [96].

Continuous education and training are essential for developing and sustaining a competent and motivated PHC workforce. Opportunities for career development, recruitment, and retention mechanisms, increasing PHC providers and introducing new cadres enhance each other. Education or short-term training equip health care providers with adequate knowledge and skills, and competency [97, 98]. Education supports the education system and PHC providers’ quality and availability; it eases the recruitment and retention and motivates health care providers. A smooth relationship between clients and PHC providers results in clients’ engagement, favourable attitudes and adherence to health care, and increased performance [99]. Mindfulness techniques effectively decreased job-related burnout among PHC providers because mindfulness training substantially decreased burnout [100].

Reviews reported the presence of appropriate scope of practice as being a promising approach to support PHC providers’ recruitment and retention process. The scope of practice reflects the procedures a health care provider can perform [101]. This allows human resource administrators for health to recruit appropriate health care providers easily. Health care providers can also practice with comfort and high job satisfaction, because of which providers would remain to work in that institution.

An appropriate work schedule is key to the recruitment and retention of PHC providers. In addition, appropriate work schedules and optimum work hours motivated health care providers [102] and prevented burnout, facilitating retention.

### Limitations

As for the study’s limitations, the current review followed the scoping review stages, during which quality appraisal was not conducted. Secondly, the included articles were only published in English.

## Conclusions

To ensure the quality and sustainability of PHC workforce, crucial aspects that need to be considered. These are preventing and avoiding burnout among primary healthcare providers, establishing or adopting a proactive system-thinking approach to keep them motivated and engaged, addressing the expected challenges and utilizing opportunities in PHC workforce development.

### Electronic supplementary material

Below is the link to the electronic supplementary material.

Supplementary Material 1

Supplementary Material 2

## Data Availability

The data set is available within this manuscript.

## Abbreviations

CHWs: Community Health Workers
PHC: Primary Health Care
UK: United Kingdom
UHC: Universal Health Coverage
UNs: United Nations
USA: United States of America
WHO: World Health Organisation
