# Supplementary material for: Barriers and strategies for primary health care workforce development: synthesis of evidence
Source: BMC Prim Care. 2024 Mar 27;25:99. doi: 10.1186/s12875-024-02336-1 (PMC10967164; doi:10.1186/s12875-024-02336-1)
Supplement: Supplementary file 1 — Supplementary Material 1 [file 12875_2024_2336_MOESM1_ESM.docx]

**Supplementary file 1: Search Strategy**

| Database | Steps | Search Strategy | Date |
| --- | --- | --- | --- |
| PubMed | 1 | ("primary health care"[Title/Abstract] OR "primary healthcare"[Title/Abstract] OR "primary care"[Title/Abstract]) AND ("work forc*"[Title/Abstract] OR "workforc*"[Title/Abstract] OR worker*[Title/Abstract] OR person*[Title/Abstract] OR professional*[Title/Abstract] OR "human resource for health"[Title/Abstract])  Then ‘Filters’ applied to Review, Systematic Review, Article language (English) | 3,838  FROM 1975 TO 2022  Date 4 July 2022 |
|  | 2 | (("primary health care"[Title/Abstract] OR "primary healthcare"[Title/Abstract] OR "primary care"[Title/Abstract]) AND ("work forc*"[Title/Abstract] OR "workforc*"[Title/Abstract] OR "worker*"[Title/Abstract] OR "person*"[Title/Abstract] OR "professional*"[Title/Abstract] OR "human resource for health"[Title/Abstract]) AND ( availability [Title/Abstract] OR accessibility [Title/Abstract] OR distribution[Title/Abstract] OR location[Title/Abstract] OR acceptability [Title/Abstract] OR coverage [Title/Abstract] OR quality [Title/Abstract] OR performance[Title/Abstract] OR education [Title/Abstract] OR training [Title/Abstract] OR development [Title/Abstract] OR deployment[Title/Abstract] OR composition[Title/Abstract] OR skill-mix[Title/Abstract] OR recruitment[Title/Abstract] OR staffing [Title/Abstract] OR turnover [Title/Abstract] OR retention[Title/Abstract] OR motivation[Title/Abstract] OR burnout[Title/Abstract] OR role*[Title/Abstract] OR responsibilit*[Title/Abstract]))  Then, Filters applied to Review, Systematic Review, and Article language (English) | 2723  FROM 1975 TO 2022  Date 4 July 2022 |
|  | Total | #1 AND #2 | 6561 |
| Web of Science | #1 | (((TI=("primary health care" )) OR TI=("primary healthcare")) OR TI=("primary care")) | 68,863 Date 4 July 2022 |
|  | #2 | ((((((TI=(workforc*)) OR TI=(work forc*)) OR TI=(worker*))) OR TI=(person*)) OR TI=(professional*)) OR TI=("human resource for health") | 594,443 Date 4 July 2022 |
|  | #3 | ((((((((((((((((((((((TI=(availability)) OR TI=(accessibility)) OR TI=(distribution)) OR TI=(location)) OR TI=(acceptability)) OR TI=(coverage)) OR TI=(quality)) OR TI=(performance)) OR TI=(education)) OR TI=(training)) OR TI=(development)) OR TI=(deployment)) OR TI=(composition)) OR TI=(skill-mix)) OR TI=(recruitment)) OR TI=(staffing)) OR TI=(turnover)) OR TI=(retention)) OR TI=(motivation)) OR TI=(burnout))) OR TI=(role)) OR TI=(responsibility) | 6,133,890  Date 4 July 2022 |
|  | #1 AND #2 refined by English and Review | #1 AND #2 and English (Languages) and Review Articles (Document Types) | 98  Date 4 July 2022  From 1990 to 2022 |
|  | #1 AND #2 AND #3 Refined by English and Review | #1 AND #2 AND #3 and English (Languages) and Review Articles (Document Types) | 20  Date 4 July 2022  From 2009 to 2022 |
|  | Total |  | 118 |
| EMBASE | 1 | ('primary health care'/exp OR 'primary healthcare' OR 'primary care') AND ('workforce'/exp OR 'work force' OR 'worker'/exp OR 'personnel'/exp OR 'human resources'/exp) AND [review]/lim AND [english]/lim | 312  Date 4 July 2022  From 1978 to 2022 |
|  | 2 | ('primary health care'/exp OR 'primary healthcare' OR 'primary care') AND ('workforce'/exp OR 'work force' OR 'worker'/exp OR 'personnel'/exp OR 'human resources'/exp) AND ('availability'/exp OR 'accessibility'/exp OR 'distribution'/exp OR 'location'/exp OR 'acceptability'/exp OR 'coverage'/exp OR 'quality'/exp OR 'performance'/exp OR 'education'/exp OR 'training'/exp OR 'development'/exp OR deployment OR composition OR 'skill mix'/exp OR 'recruitment'/exp OR staffing OR 'turnover rate'/exp OR 'retention'/exp OR 'motivation'/exp OR 'burnout'/exp OR 'role'/exp OR 'responsibility'/exp) AND [review]/lim AND [english]/lim | 157  Date 4 July 2022  From 1985 to 2022 |
|  | Total |  | 469 |
