# Supplementary material for: Barriers and strategies for primary health care workforce development: synthesis of evidence
Source: BMC Prim Care. 2024 Mar 27;25:99. doi: 10.1186/s12875-024-02336-1 (PMC10967164; doi:10.1186/s12875-024-02336-1)
Supplement: Supplementary file 2 — Supplementary Material 2 [file 12875_2024_2336_MOESM2_ESM.docx]

Supplementary file 2: Characteristics of included articles

| Citation | Countries discussed | Number of studies reviewed | Types of review | Studied issues | Domain |
| --- | --- | --- | --- | --- | --- |
| Colon-gonzalez mc. 2015 | Central America | 20 | Narrative review | Rural health medical education | Education |
| Costa GDD. 2019 | European and North American continent | 13 | Integrative review | Dementia training for PHC professionals | Education |
| Howarth M. 2006 | United States of America | 24 | Literature review | Educational needs for integrated care | Education |
| Kelley LT. 2020 | Not specified | 37 | Systematic review | Continuing professional development for PHC Providers in Palliative and End-of-Life Care | Education |
| Lord J. 2003 | United States of America | Not specified | Review | Future of PHC education | Education |
| Mccalman J. 2019 | Canada, Australia, New Zealand and United States of America | 28 | Scoping review | Indigenous PHC workforce development | Distribution |
| Nayahangan LJ. 2021 | Not specified | 46 | Systematic review | Training and education of healthcare  workers | Education |
| Duran-Niño EY 2021 | Not specified | 40 | Narrative review | Nursing professionals training | Education |
| Bennett KL. 2010 | United States of America | 6 | Systematic review | Finding, recruiting, and sustaining the future PHC physician workforce | Recruitment and retention |
| Deroy S. 2019 | Australia | 26 | Literature review | Aboriginal health and wellbeing staff | Retention/turnover |
| Feeley TH. 2003 | Not reported | Not specified | Review | Rural practicing physicians | Retention/turnover |
| Haggerty TS. 2013 | United States of America | Not specified | Review | Physician wellness in rural Americas | Retention/turnover |
| Halcomb E. 2018 | Worldwide | 20 | Integrative review | Career intention of registered nurse | Retention/turnover |
| He R. 2020 | China | 16 | Systematic review and meta-analysis | Turnover intention of PHC workers | Retention/turnover |
| Heidi S. 2020 | Canada | 5 | Systematic review | Recruitment and retention in rural Canada | Recruitment and retention |
| Kueakomoldej S. 2021 | United States of America | 22 | Scoping review | Recruitment and retention of primary care nurse practitioners in underserved areas | Recruitment and retention |
| Long L. 2020 | United Kingdom | 6 | Systematic review | Primary care doctors leave direct patient care | Retention/turnover |
| Marchand C. 20-2017 | Organisation for Economic Cooperation and Development countries | 138 | Systematic review | General practitioner recruitment and retention | Recruitment and retention |
| Couch A. 2021 | Not specified | 22 | Systematic review | Factors of workplace location on allied health professional | Recruitment and retention |
| Ogden J. 2020 | Australia | 27 | Systematic review and meta-analysis | General practitioner in rural practice | Recruitment and retention |
| Parlier AB. 2018 | Not specified | 83 | Narrative review | Rural primary care physicians | Recruitment and retention |
| Russell DJ. 2017 | Australia | 8 | Review | Rural Australian PHC worker | Retention/turnover |
| Storey C. 2009 | United Kingdom | Not specified | Literature review | Nurse in PHC and CHC | Retention/turnover |
| Terry D. 2021 | Australia, Ukraine, Canada, United States of America, New Zealand | 13 | Systematic review | Rural pharmacist | Recruitment and retention |
| Verma P. 2016 | High-income countries | 51 | Systematic review | Primary care doctors | Recruitment and retention |
| Stochkendahl MJ. 2019 | Not specified | 90 | Review | Chiropractic workforce | Distribution |
| Blay N. 2021 | Australia | 25 | Systematic review | Community nurse | Distribution |
| Franco CM. 2021 | Not specified | 69 | Integrative review | Health workforce in primary care in rural areas | Distribution |
| Goodfellow A. 2016 | United States of America | 72 | Systematic review | Physician location to underserved area | Distribution |
| O’Connor TM. 2007 | United States of America | Not specified | Systematic review | The physician assistant model | Distribution |
| Safi N. 2018 | Afghanistan | Not specified | Literature review | Health workforce shortages and maldistribution | Distribution |
| Poghosyan L. 2021 | United States of America | 14 | Literature review | Growing primary care nurse practitioner workforce | Role and responsibility |
| Eaton G. 2021 | United Kingdom, Australia, Canada, and Americas | 205 | Realist review | Role of the paramedic in primary care | Role and responsibility |
| Henry LR. 2011 | United States of America | 28 | Systematic review | Role of physician assistant on rural health care | Role and responsibility |
| Hooker RS. 2012 | United States of America, Canada, United Kingdom, Australia, Saudi Arabia | 15 | Literature review | Contributions of physician assistants in PHC systems | Availability |
| Morely JE. 2017 | Canada, Japan, Germany, Italy, United States of America, France, United Kingdom | 48 | Systematic review | Enhancing the Role of the Primary Health Care Professional in Preventing Functional Decline | Role and responsibility |
| Sheringham J. 2021 | United States of America, Canada, United Kingdom | 15 | Systematic review | Physician associate/assistant contributions to cancer diagnosis in primary care | Performance |
| Torrens C. 2019 | Asia, Europe, Americas, Oceania, and Multiple regions | 54 | Scoping review | Advanced nurse practitioner role | Role and responsibility |
| Yellamaty V. 2019 | Australia | 59 | Integrative review | General practitioners with special interests’ role and impact | Performance |
| Vasan A. 2017 | Low-and middle-income countries | 40 | Scoping review | Support and performance improvement for PHC workers | Performance |
| Ballard M. 2016 | Low-and middle-income countries | 14 | Systematic review | Interventions improve performance of CHWs | Performance |
| Jeyaraman MM. 2021 | North America, Europe, Asia, Australia, Middle East | 40 | Systematic review and meta-analysis | Impact of employing primary healthcare  professionals in emergency department | Performance |
| Kok MC. 2014 | Low-and middle-income countries | 140 | Systematic review | Factors influence performance of CHWs | Performance |
| Martı´nez-Gonza´lez NA. 2014 | United Kingdom, Netherlands, United States of America, Russia and South Africa | 12 | Systematic review and meta-analysis | Effects of physician-nurse substitution on performance | Performance |
| RobertshawL. 2017 | High-income countries | 21 | Systematic review | Challenges and facilitators for PHC professions for refugees | Performance |
| Desborough J. 2011 | United Kingdom | 13 | Integrative review | Nurse-led PHC walk-in centres | Performance |
| Williams DM. 2010 | United Kingdom | 26 | Systematic review | Skill-Mix and Service Transfer in Primary Care Settings | performance |
| Howatson A. 2015 | New Zealand | 21 | Systematic review | Dietitians’ contribution to PHC workforce | Performance |
| Khangura JK. 2012 | United Kingdom | 3 | Review | Primary care professionals non-urgent care in hospital emergency | Performance |
| Leong SL. 2021 | United States of America, United Kingdom, Netherlands, Russia, Canada, Australia, low and middle-income countries | 21 | Umbrella review | Task shift from physician to allied health worker | Performance |
| Xue Y. 2015 | Not specified | 15 | Systematic review | Impact of state nurse practitioner scope-of-practice regulation on health workforce, access to care, utilisation, cost | Performance |
| Källander K. 2013 | Low-and middle-income countries | Not specified | Review | Mobile Health for performance and retention of CHWs | Performance & retention |
| Stephen C. 2017 | United Kingdom, Australia, Holland, New Zealand | 11 | Integrative review | Nurse-lead PHC interventions | Acceptability |
| Scope A. 2021 | United Kingdom | 9 | Systematic review | primary or community based-behavioural interventions | Acceptability |
| Islam MM. 2012 | Greece, United Kingdom, Malaysia, Australia, Canada, United States of America, Finland, Ireland, Sweden, Nepal, Iran | 35 | Narrative review | Acceptability of PHC outlets on injecting drug users | Acceptability |
| Behar E.2018 | United States of America | 17 | Systematic review | Acceptability of Naloxone Prescribing | Acceptability |
| Shipton L. 2017 | Low-and middle-income countries | 17 | Systematic review | Community health workers | Motivation |
| Campbell N. 2012 | Australia, Canada and America | 35 | Literature review | Rural and remote allied health professional | Motivation |
| Gadsden T. 2021 | Low-and middle-income countries | 12 | Systematic review | Community health workers | Motivation and performance |
| Li H. 2019 | China | 36 | Systematic review and meta-analysis | Primary care workers | Motivation and performance |
| Abraham CM. 2020 | United States of America | 21 | Systematic review | Primary care workers | Burnout |
| Dugani S.2018 | Low-and middle-income countries | 20 | Systematic review | Frontline PHC provider | Burnout |
| Garcia GPA.2018 | Not specified | 14 | Integrative review | PHC Provider | Burnout |
| Monsalve-Reyes CS. 2018 | Not specified | 8 | Systematic review and meta-analysis | Primary care nurse | Burnout |
| Pérez-Francisco DH.2020 | Not specified | 45 | Integrative review | Primary care nurse | Burnout |
| Salvado M. 2021 | Not specified | 16 | Systematic review and meta-analysis | Primary care professional | Burnout |
| Wright T. 2022 | Low-and middle-income countries | 60 | Systematic review and meta-analysis | PHC professionals | Burnout |
| Davis MM. 2014 | Not specified | 15 | Systematic review | Clinician and Staff Views on the Acceptability of Incorporating Remote Monitoring Technology into Primary Care | Acceptability |
